# Detecting epigenetic motifs in low coverage and metagenomics settings

**DOI:** 10.1186/1471-2105-15-S9-S16

**Published:** 2014-09-10

**Authors:** Noam D Beckmann, Sashank Karri, Gang Fang, Ali Bashir

**Affiliations:** 1 Institute for Genomics and Multiscale Biology & Department of Genetics and Genomic Sciences, Icahn School of Medicine at Mount Sinai, One Gustave L. Levy Place, New York, NY, USA

## Abstract

**Background:**

It has recently become possible to rapidly and accurately detect epigenetic signatures in bacterial genomes using third generation sequencing data. Monitoring the speed at which a single polymerase inserts a base in the read strand enables one to infer whether a modification is present at that specific site on the template strand. These sites can be challenging to detect in the absence of high coverage and reliable reference genomes.

**Methods:**

Here we provide a new method for detecting epigenetic motifs in bacteria on datasets with low-coverage, with incomplete references, and with mixed samples (i.e. metagenomic data). Our approach treats motif inference as a kmer comparison problem. First, genomes (or contigs) are deconstructed into kmers. Then, native genome-wide distributions of interpulse durations (IPDs) for kmers are compared with corresponding whole genome amplified (WGA, modification free) IPD distributions using log likelihood ratios. Finally, kmers are ranked and greedily selected by iteratively correcting for sequences within a particular kmer's neighborhood.

**Conclusions:**

Our method can detect multiple types of modifications, even at very low-coverage and in the presence of mixed genomes. Additionally, we are able to predict modified motifs when genomes with "neighbor" modified motifs exist within the sample. Lastly, we show that these motifs can provide an alternative source of information by which to cluster metagenomics contigs and that iterative refinement on these clustered contigs can further improve both sensitivity and specificity of motif detection.

**Availability:**

https://github.com/alibashir/EMMCKmer

## Background

DNA modification can occur in a wide variety of living organisms, from bacteriophages [1,2] to prokaryotes [3,4] and eukaryotes [5]. They range from directed and controlled modifications to more irregular damage events [6,7]. These modifications can trigger a wide variety of functions, such as origin of replication (oriC) firing in *E. coli *[8,9] and gene silencing in humans [10]. DNA methylation is so far the best understood and most well characterized of modification events[4,8,9,11]. In eukaryotes, DNA methylation has been most commonly seen on cytosine at position 5 (m5C) [10,12]. In bacteria the 4^th ^or 5^th ^positions of C can be methylated (m4C, m5C), as well as the 6^th ^position in Adenine bases (m6A) [3,4,8,11].

Multiple methods around high-throughput sequencing technologies have emerged for genome-wide detection of epigenetic events, the most common being bisulfite sequencing. There, DNA is treated with a bisulfite reagent that converts unmethylated Cytosine to Uracil, but does not affect m5C bases, and is then amplified. After amplification, high-throughput sequencing (typically short-reads generated on the Illumina platform) is performed on the library to identify positions of m5C in the genome [13]. This method, while extremely high-throughput and cost-effective, is limited in the scope of modifications it can detect. Only specific forms of methylation, that can be enzymatically converted and mapped to well-defined references, are targeted.

Single Molecule Real Time (SMRT) sequencing from Pacific Biosciences is a third-generation sequencing technology that monitors a polymerase in real time as it sequences a single fragment of DNA. It has the unique capability to directly detect native epigenetic modifications by monitoring the time between base incorporations (or inter-pulse durations, IPDs). In short, a modification (such as methylation) causes variation (termed "kinetic variation") in the rate at which a polymerase reads the template. Flusberg et al. were the first to use synthetic DNA to demonstrate that kinetic variations, as recorded by SMRT sequencing, are associated with distinct DNA methylations [14]. In particular, m6A and hydroxymethyl-5-Cytosine (hm5C) were shown to be associated with reliable, robust kinetic signatures. Advanced statistical methods were also proposed to more accurately detect DNA modifications when including a conditional random field (CRF) based framework [15] and a hierarchical Bayesian based framework [16]. The latter also explored the dependency of IPD on local sequence context. Several genome-wide studies applied SMRT sequencing to real bacterial genomic DNA and characterized the methylomes of multiple species and strains [3,4].

These studies have revealed that regulatory roles of bacterial methylations on transcription were more extensive than anticipated. Specifically, by comparing a wild type *E. Coli *strain that caused 2011 German outbreak with a mutant strain without a restriction modification system, Fang et al. showed that more than one fifth of all genes were significantly differentially expressed [3]. The connection between differential methylation and differential gene expression was implicated in cell-cycle regulation [11]. Motivated by such findings, an increasing number of SMRT sequencing epigenetic studies are now being performed on a diverse collection of bacterial species.

Though the precise methods for detecting DNA modifications at the motif level vary between studies, the fundamental process follows a regular pattern:

Sequence a genome to at least 10X coverage (usually higher) [17]

Map reads to a reference genome and identify IPD distributions at each position

At each position compare IPD distributions of native sequence to modification free sequences (either whole genome amplified (WGA) or *in silico *control sequences) to characterize significant deviations from expectation

Rank-order modified positions by significance in the genome

Pass sequences surrounding the highest ranking positions to a motif finding program (such as MEME [18]) to identify significantly overrepresented motifs

Iteratively remove significant motif sequences from ranked list, and rerun Step 5 until no new significant motif appears.

This methodology allows for high-fidelity detection of motifs, but is limited in several ways. First, it entails at least moderate sequencing depth across a clonal genome. Very low-coverage could lead to many missed motifs given the exponential like distribution of IPDs, and the high sequencing error rate of the platform [19]. Similarly, if the samples were mixed, background noise could pollute the true signal and lead to false negatives. Second, these studies employ reference genomes (or construct reference sequences via deep sequencing). However, in some species/strains, such references are not readily available. Furthermore, in a metagenomics context one may not even know *a priori *what strains are being sequenced. Additionally, the relative coverage of genomes in a sample may vary tremendously. Detecting motifs in this less-controlled setting has, to date, been avoided.

Here, we describe a novel approach for detecting epigenetic motifs without the need for high-coverage, clonal samples or complete references. We assess the accuracy of our method by testing it on six published bacterial genomes, with matched native and WGA SMRT sequencing data. Our results show that we can recover previously discovered motifs, with N(6)-methyladenine ((m6)A) and N(4)-methylcytosine ((m4)C) modifications, in both low-coverage and high-contamination scenarios. Additionally, we show the potential for metagenomics applications by synthetically mixing three strains and recovering many motifs, even when the motif sequences overlap heavily between the genomes of interest. Then, in a paired short-read metagenomics simulation we indicate our ability to not only recover motifs but, also, cluster fragmented contigs (by species) without additional genomic features. Lastly, we show that motif predictions can be iteratively refined using these predicted clusters.

## Methods

**Figure 1:**
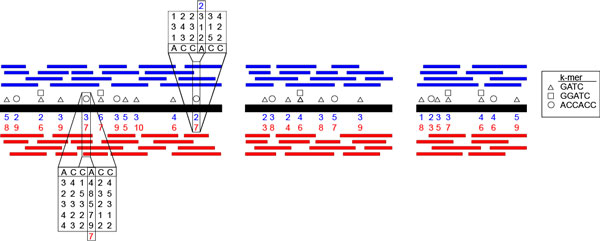
**Overview of Kmer Grouping of Motifs**. Sequences are mapped to a genome or contig set (black). The median native (red) and WGA (blue) IPD is taken at each position. Sequences are then deconstructed into kmers corresponding to potential motifs (indicated by circles, rectangles, and triangles). The distributions of native and WGA median IPDs for each motif are used to generate its LLR.

Figure 1 shows a schematic representation of the first steps of our analysis. We show a reference genome (black) with modified positions indicated by squares, circles and triangles. When a base is modified, one expects native reads (red) to contain longer IPDs at that position than WGA reads (blue). In this example, the distribution of IPDs corresponding to the second 'A' of "ACCACC" appears to be, on average, longer in native reads than corresponding WGA reads. In order to reduce the high variability of individual IPD reads at a given genomic (or contig) position and improve computational efficiency, we maintain only the median IPD at each position. The median is selected in lieu of the mean because it is more stable to outliers. Selecting a single value (median) at each position also ensures that each occurrence of a motif in the genome is equally weighted. Without this, sampling artifacts between WGA and native sequences could lead to spurious calls when a given context is sampled disproportionality, as often occurs in low-coverage situations.

The approach then consists of three distinct components. First, we compute log likelihood ratios (LLRs) on distributions of median IPDs for individual kmers between native and WGA datasets. Next, we use these LLRs to predict motifs that are likely to be modified using a two-phase algorithm. Finally, when dealing with metagenomic datasets with no reference, we perform an iterative motif prediction/clustering approach to improve the sensitivity and specificity of our motif predictions.

### LLR distributions on Kmers

In the first step, we construct a kmer table representing all existing kmers observed in the reference genome (or contigs). To date, most modified motifs detected using SMRT sequencing have sizes that ranged from 4-6 base pairs [4]. Though longer motifs have been observed, they often are in the form of dyad motifs that contain degenerate bases ('N's) between two shorter conserved motifs [3,4]. As such, we enforce that 4≤k≤7, where *k *is the length of a motif. This makes the maximal size of our kmer table: $\underset{4\leq k\leq 7}{\sum }k\cdot 4^{k}$. Let *X *be the set of all motifs in our sample, and the length *k *of a motif, *X_i_*, be given by $\left|X_{i}\right|$. Let $X_{i,S}$ represent the set of observed median native IPDs for sample *S *(corresponding to some set of reference sequences), and $X_{i,S^{\prime }}$ represent the set of median WGA IPDs in *S*. For each $X_{i,S}$ we calculate the LLR $\left(X_{i}.LLR\right)$ using $X_{i,S^{\prime }}$ as the null distribution. We assume that the log transformed median IPDs roughly follow a normal distribution, following the site-specific model LLR statistic used in the *E. coli *dataset referred to in Table 1 [3]. As context effects play a more dramatic role when grouping values across sites, the normality assumption may be less robust in this scenario.

**Table 1:** Previously identified motifs

Species	Motif	% Modified	Study
*E. coli*	G ^m6^ATC	94.1	Fang 2012
*E. coli*	ACC ^m6^ACC	93.2	Fang 2012
*E. coli*	CTGC^m6^AG	96.3	Fang 2012
*G. metallireducens*	G ^m6^ATCC	99.2	Murray 2012
*G. metallireducens*	GG ^m6^ATC	98.7	Murray 2012
*G. metallireducens*	TCC ^m6^AGG	98.2	Murray 2012
*B. Cereus *ATCC 10987	CGA ^m6^AG	93.3	Murray 2012
*B. Cereus *ATCC 10987	A ^m4^CGGC	33.8	Murray 2012
*B. Cereus *ATCC 10987	TGC ^m4^CG	47.5	Murray 2012
*C. Jejuni *81-176	RA ^m6^ATTY	99.0	Murray 2012
*C. Jejuni *81-176	GCA ^m6^AGG	97.7	Murray 2012
*C. Jejuni *81-176	GGRC ^m6^A	97.6	Murray 2012
*C. Jejuni *NCTC 11168	RA ^m6^ATTY	99.2	Murray 2012
*C. Jejuni *NCTC 11168	GKA ^m6^AYG	98.2	Murray 2012
*C. Salexigens*	RG ^m6^ATCY	76.5	Murray 2012

Motifs from previous studies [3,4] used to validate the new method. Degenerate bases are identified via corresponding IUPAC symbols. The "RG ^m6^ATCY" motif consensus corresponds to 4 distinct motifs.

After LLRs have been computed for each motif, a significance cutoff is calculated. In principle, the LLR distribution should follow a chi-squared distribution [20] ($\chi^{2}$). However, the LLRs are not completely independent from one another. For example, motifs of size *k *are substrings of motifs of size *k*+1. We term these motifs of size *k *'parents', the corresponding motifs of size *k+1 *'children' and the set of parent and children for a given motif *X_i_* as 'neighbors'. To reduce the dependence between neighbors, we evaluate separately LLR significance for each *X_i_* only with motifs $X_{j}\in X\text{ such that }|X_{j}|=|X_{i}|$. Unfortunately, this does not break all dependencies; for each *k*, motifs may contain overlapping prefix or suffix strings. Additional heuristics are employed to address this in the next section.

In practice, it was observed that fitting LLR distribution to a $\chi^{2}$ overestimated significance, leading to a large number of false positives. Instead, we choose to fit LLRs using a gamma distribution ( Γ, which, in addition to being more flexible, permits chi-squared distribution fitting, as all $\chi^{2}$ exist as a special case of  Γ. Modifications are believed to cause increases in observed IPDs, and LLRs derived from motifs where the WGA IPD is larger than the native IPD are considered noise. The max such LLR value is determined; all motifs with LLRs less than either this value (or less than 99% of all remaining LLRs) are included in the gamma distribution fit, in order to mitigate fitting of outliers. Outlier motifs are then identified by computing $p_{X_{i}}=1-CDF\left(\Gamma \left({\text{X}}_{\text{i}}\right)\right)$, and comparing to a Bonferroni corrected significance cutoff, $p_{\gamma k}$, where $p_{\gamma k}=\frac{t_{\gamma }}{k4^{k}}$. In practice, motifs can be compared after adjusting the survival probabilities for each motif *X_i_*, for the correction factor ($X_{i}.GammaCorr=p_{X_{i}}\cdot k4^{k}$).

### Obtaining significant motifs

Given the complex neighborhood of each motif, one cannot simply take all motifs that pass the Bonferroni corrected cutoff as significant. Many motifs that are simply parents or children of a true motif would appear significant. However, we cannot simply ignore all neighbors of a motif *X_i _*because its parents, children, or "shifts" (where the prefix or suffix is shared between kmers of the same length) may be significant in their own right. For example, in Table 1, the modified *E. coli "*GATC" motif is parent of two modified motifs in *G. metallireducens *("GGATC" and "GATCC") which are, in turn, parents of four modified motifs in *C. salexigens*).

To address these considerations, we developed a two-phase algorithm (Algorithm 1). To summarize, motifs are first ranked by their LLRs and a set of independent motifs that pass the significance threshold is created (Algorithm 2). Neighbors of these significant motifs are then identified and an additional significance evaluation is performed for neighbors of Phase 1 motifs (Algorithm 3).

**Algorithm 1 **MotifDetector(*X*,*t*_γ_*,t_N_*)

1: *O *= MotifDetectPhase1 $\left(X,t_{\gamma },0̸\right)$

2: **for ***X_m _*∈ *O ***do**

3:     *N_Xm _*= Neighbors(*X*_m_)

4:     *μ *= MeanLLR(*N_Xm_*) # *Mean LLR for neighbors*

5:     σ = StDevLLR(*N_Xm_*) # *Standard Deviation of LLR for neighbors*

6:     **for ***N_i _*∈ *N_Xm _***do**

7:         $N_{i}.\text{NormalProb}=\frac{1}{\sigma \sqrt{2\pi }}\int_{x}^{\infty }e^{\frac{-{\left(t-\mu \right)}^{2}}{2\sigma^{2}}}dt$

8:     **end for**

9: **end for**

10: **return **MotifDetectPhase2 (*N*,*t_N_*, *t*_γ_,*O*)

Phase 1 is detailed by Algorithm 2. We begin by ranking all motifs by their LLR. The motif with the largest LLR score, *X_m _* is tested to see if its adjusted probability passes the gamma probability cutoff, $t_{\gamma }$ (set to $10^{-6}$). If *X_m _*does not pass the cutoff, the algorithm terminates. If *X_m _*passes this cutoff it attempts to evaluate the neighborhood of a motif. First, it checks if a parent of *X_m _*has a higher LLR than *X_m _*itself (suggesting that motif is not truly driving the observed deviation). If no such parent exists then *X_m _*is added to the set of true motifs, *O*, and its neighbors are eliminated from further evaluation in Phase 1. Whether or not such a parent is found, the algorithm is recursively called on a new set $X^{\prime }=X\backslash X_{m}$.

After Phase 1 is complete, the neighbors of motifs in *O *are evaluated for significance. The underlying assumption is that if a neighbor, $N_{i}$ of a true motif, *X_m _*, is truly significant, then its LLR should not only be an outlier within the distribution of all kmers (since this is expected given that a subset of their observations are driven by *X_m _*) they should also be significant relative to the set of all neighbors of *X_m _*, $N_{X_{m}}$, of the same kmer size (the set of neighbors $N_{j}\in N_{X_{m}}\text{ such that }|N_{j}|=|N_{i}|)$. We make the naïve assumption that this distribution of LLRs neighbors should be roughly normal for any motif length *k*, and thus calculate the probability that a motif $N_{i}$ is an outlier relative to this distribution, $N_{i}.NormalProb$. Once this probability is computed, we pass the neighbors to the Phase 2 algorithm described by Algorithm 3. The algorithm recursively selects the most significant motif, $N_{i}$, based on $N_{i}.NormalProb$. Similar to Phase 1, if this value does not pass a Bonferroni corrected significance cutoff for neighbors, $t_{N}$ (set to  10 standard deviations), the algorithm terminates. If the algorithm passes this cutoff as well as the previous gamma probability cutoff it is selected as a true motif. Independent of whether the motif is designated as significant, the algorithm is recursively called on the remaining set of neighbors. These motifs are combined with those discovered in Phase 1 to yield the final set of motif predictions.

**Algorithm 2 **MotifDetectPhase1 (*X*,*t*_γ_*,O*)

1: m = arg max*_i_*(*X_i_.LLR*) # *find most significant motif*

2: **if ***X_m_*.*GammaCorr *<*t*_γ _**then**

3:     **return ***O *# *if X_m _fails t*_γ _*threshold, terminate*

4: **end if**

5: **if ***X_m_*.*LLR *> max_*j∈Parents*(*Xm*)_(*X_j_.LLR*) **then**

6:     *X = X\ *Neighbors(*X_m_*) *# remove neighbors of X_m _from X*

7:     *O = O *∪ *X_m _# add X_m _to significant motifs*

8: **end if**

9: **return **MotifDetectPhase1 (*X \ X_m_,t*_γ_*,O*) *# get next most significant motif*

**Algorithm 3 **MotifDetectPhase2 (*N,t_N_*,*t*_γ_*,O*)

1: *m *= arg min*_i_*(*N_i_.NormalProb*) *# find most significantly deviated neighbors*

2: **if ***N_m_.NormalProb < t_N _***then**

3:     **return ***O # if N_m _fails neighbor threshold, terminate*

4: **end if**

5: **if ***N_m_.GammaCorr < t*_γ_,**then**

6:     *O *= *O *∪ *N_m _# if N_m _passes t*_γ _*threshold, it is significant*

7: **end if**

8: **return **MotifDetectPhase2 (*N *\ *N_m_*,*t_N_*, *t*_γ_*,O*) *# get next most significant motif*

### Clustering contigs by significant Kmers and improving motif resolution

Metagenomic datasets provide additional challenges that necessitate extensions to the single genome algorithm presented above. In most metagenomic samples, one does not know the constituent genomes a priori; instead one uses the Metagenomic reads to assemble contigs derived from the mix of bacterial genomes in the sample. Also, one might expect that the genomes do not share (or only partially share) motifs. Algorithm 1 would only be expected to identify motifs that are significant across the entire mixture. Though it could likely detect some motifs, it would potentially miss many true motifs that were either in genomes with low abundance or had low kinetic variation. In principle, the initial set of significant motifs could be used as a feature set for clustering contigs. If two contigs have similar epigenetic profiles, one might expect that they are likely to belong to the same (or similar) genome(s).

Consider our set *O *of significant motifs discovered by the previous two algorithms. Let $X_{i,S_{c}}$ be the set of median IPDs for motif *X_i _*in contig *c *and $X_{i,S_{c}^{\prime }}$ be the corresponding set of WGA IPDs. For each contig, *c*, we create a vector, $V^{c}$ of size |O|, where each value, $V_{j}^{c}$ corresponds to the mean ratio of the median IPD per positions, $V_{j}^{c}=\frac{\bar{O_{j,S_{c}}}}{\bar{O_{j,S_{c}^{\prime }}}},$ when available. We can now cluster contigs by their distances between this representative vector. Here, we use K-means (with K = 3) for clustering with Euclidean distance as our metric. After clustering, new LLR values are calculated for each cluster, *C*, of contigs. The motif detection algorithm is then run independently on each of the clustered contig sets. This enables one to detect distinct, potentially non-overlapping motifs, within each cluster, leading to improved sensitivity and higher specificity within each cluster.

## Results

We assessed our ability to detect methylation on published SMRT sequencing data from six different genomes showed in Table 1 [3,4]. Runtimes for the full datasets are seen in Supplemental Table 1 (additional file 1) These particular datasets were selected as they had matched native and WGA sequencing data for each genome. Additionally, the similarity of motifs (specifically in the overlap of "GATC" like motifs) in three of the genomes presents a particularly challenging test case for our method, given the complex neighborhood relationships between kmers within and across the 6 genomes. We ran two different types of simulations on the combined dataset. First, we examined each genome independently at differing coverages and levels of background noise. Second, we examined mixtures of the three genomes that share similar motifs at different ratios.

### Single genome simulation

**Figure 2:**
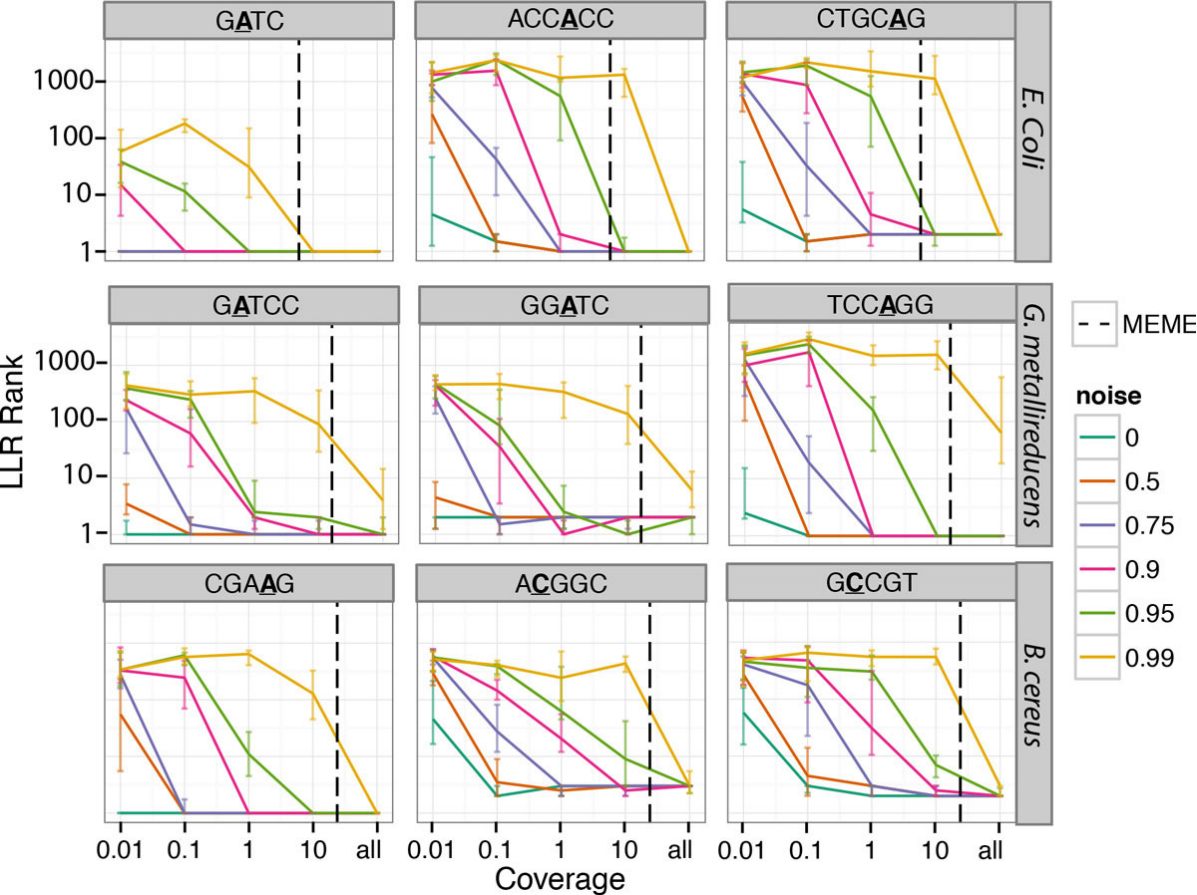
**LLR ranking of true Kmers**. Each kmer is ranked by its LLR score for multiple coverages and levels of "noise" (colors). The x-axis represents the coverage after downsampling (where "all" represents the entire dataset) and the y-axis the rank of the associated LLR value. Each point represents the median of 10 simulations, with upper and lower quartile bounds given by error-bars. Vertical black dashed lines correspond to the lowest coverage in which a majority of MEME runs (5 iterations) detect *any *true motif for the corresponding genome. The modified position per kmer is highlighted in each subplot title. Children of "GATC" have been removed from *E. coli *plots and children of "GGATC" and "GATCC" have been removed from *G. metallireducens *plots.

For each genome we varied coverage from 0.01X to the entire available coverage and noise levels from 0% to 99% contaminant reads. In each dataset, noise was introduced by mixing a corresponding proportion of WGA reads with the downsampled native reads to create the simulated "native" dataset. Figure 2 (Supplemental Figure 1) (additional file 1) shows the absolute rank of true motifs, across the spectrum of sequencing depth and WGA contamination.

Except for highly degenerate motifs, at 10X coverage nearly every true motif is consistently observed within the top 5 LLRs (when controlling for significant neighbors of higher ranked motifs, Figure 2), even when 90% of the total sequencing data is WGA contamination. Most motifs are highly ranked even when 95% of the sequencing data is non-native sequence (GATC is detectable at 10X coverage even in 99% background noise). Additionally, most motifs can be detected well below 1X coverage in low noise scenarios.

**Figure 3:**
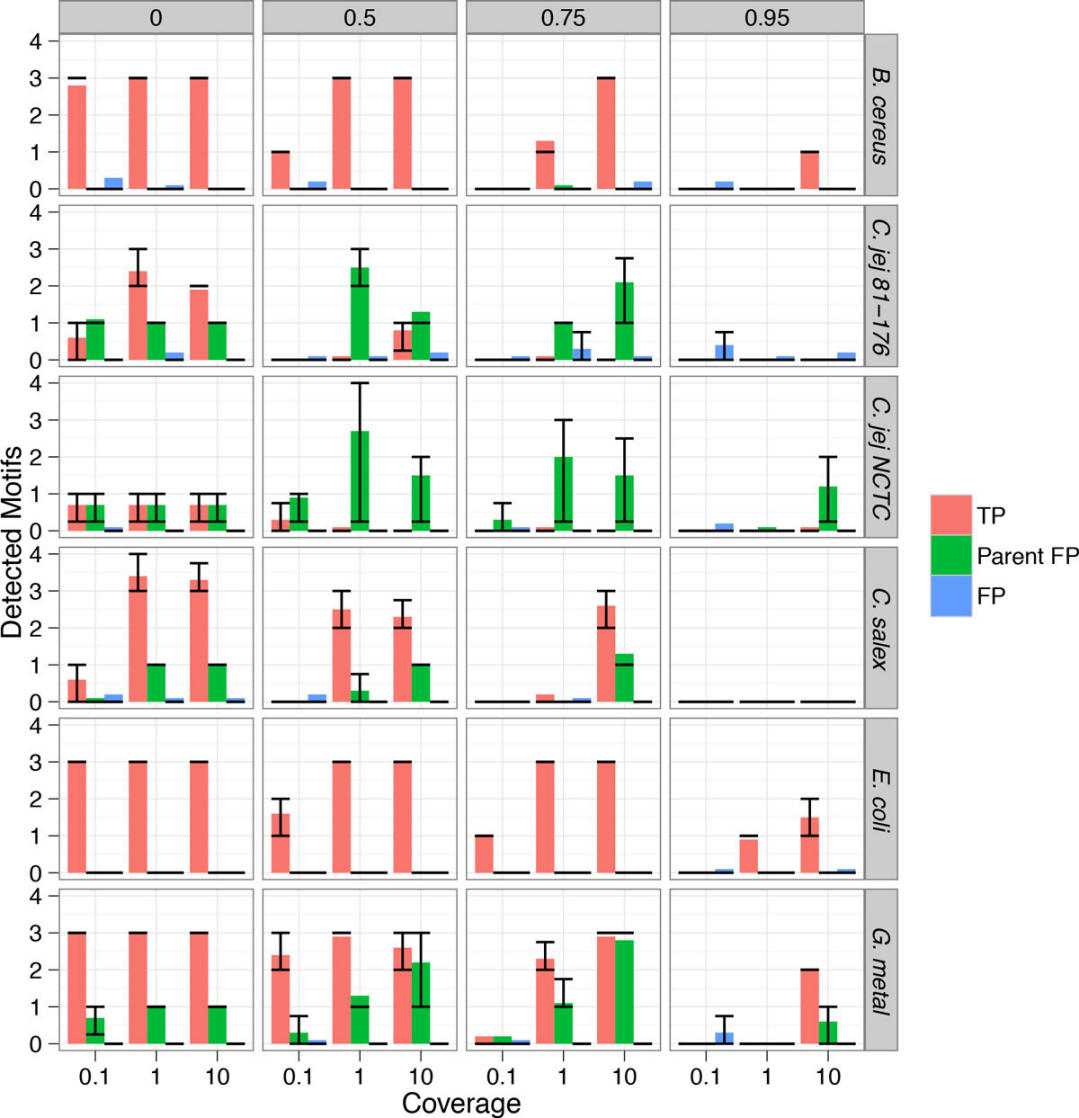
**Significant motifs returned by two-phase algorithm**. For each simulation in Figure 2, we applied the two-phase algorithm to determine a set of significant motifs. The x-axis corresponds to the coverage after downsampling and the y-axis to the number of detected motifs. True positives counts (red) correspond to detection of motifs as described in Table 1. The total (and consensus) motifs for each are: 3 (3) for *E. coli*, 3 (3) for *G. metallireducens*, 4 (1) for *C. salexigens*, 3 (3) for *B. cereus*, 7 (3) for *C. jejuni *81-176, 8 (2) for *C.jejuni *NCTC 11168). Parent false positives counts (green) are the parents of true motifs all other false positives are denoted by blue. Each row of subplots corresponds to a specific bacteria. Each column of subplots corresponds to the simulated WGA noise fraction. Error bars correspond to upper and lower quartiles.

The ranking information is not sufficient to determine whether a motif is real, given that different samples may contain varying numbers of true motifs. Moreover, complex relationships between neighbors further complicate assessment of "true" motifs based purely on a ranking scheme. To address this we applied our two-phase algorithm on the same datasets across all kmer sizes (Figure 3). In low noise scenarios we detect all (or nearly all) motifs with few false positives with the exception of *C. jejuni *strains, where only partial detection is observed.

Even in low-coverage scenarios the methods are often able to correctly recall true motifs. In the case of *E.coli *and *G. metallireducens*, this stays true, at 1X coverage, even when 75% of the sequencing data is contamination (at 10X coverage they are able to maintain nearly all true positives even at 90% contamination). Additionally, in most cases where a true motif is detected, it is higher ranked (in either Phase 2 or Phase 3) than any of the false positive motifs (data not shown). Notably, in simulation, greater or equal than 6X coverage was required to detect our strongest motif, "GATC" using the MEME algorithm [18] (Figure 2) even without any added noise.

The nature of the false positives is also interesting. In the case of *C. salexigens *the false positive sets always contain "GATC", a parent motif for all four of the true motifs. Notably, when it appears, it is always present with a higher LLR than "AGATCT", the weakest of the four motifs. Additionally, the second "false positive" (when present) is usually "CCAC" a portion of a known dyad motif ("CC ^m6^ACN6CTC") in *C. salexigens *[4]. This suggests that the method could be readily extended to dyads by examining these near true hits. In addition, at lower significance cutoffs *E. coli *sometimes reports false positives with respect to neighbors of "GATC", suggesting potential improvements to the neighborhood significance calculation, though some of these may be accounted for by the "GATC" like dyad motif "(A/G)TC ^m6^AN8GTGG" [3] (data not shown).

### Metagenomics simulation

Metagenomic datasets were derived from simulated mixtures of three genomes, *E. coli, G. metallireducens *and *C. salexigens*, by sampling reads at different levels of coverage from each sample. In addition to having detectable motifs (Figure 2), these genomes were selected given their shared "GATC" motif root, which could prove confounding in a metagenomics context. For consistency we fixed the total absolute PacBio sequencing depth (across the mixture) to be 20X. We then varied the proportion of one of the genomes from 1-18X, while splitting the remaining coverage evenly among the two other genomes. For each dataset, we also simulated a set of Illumina reads (2x100 bp reads with 500 bp inserts and 1% error, using wgsim [21]) at 25 times the coverage of the corresponding PacBio read. The reads were then assembled using MetaVelvet and Velvet (using a kmer of size 60 and setting 500 bp as the expected insert length) [22,23]. The higher sequencing depth for Illumina was thought to be natural given its substantially lower per-base cost and the necessity of having reasonable depth of each genome to perform any sort of metagenomic assembly.

**Figure 4:**
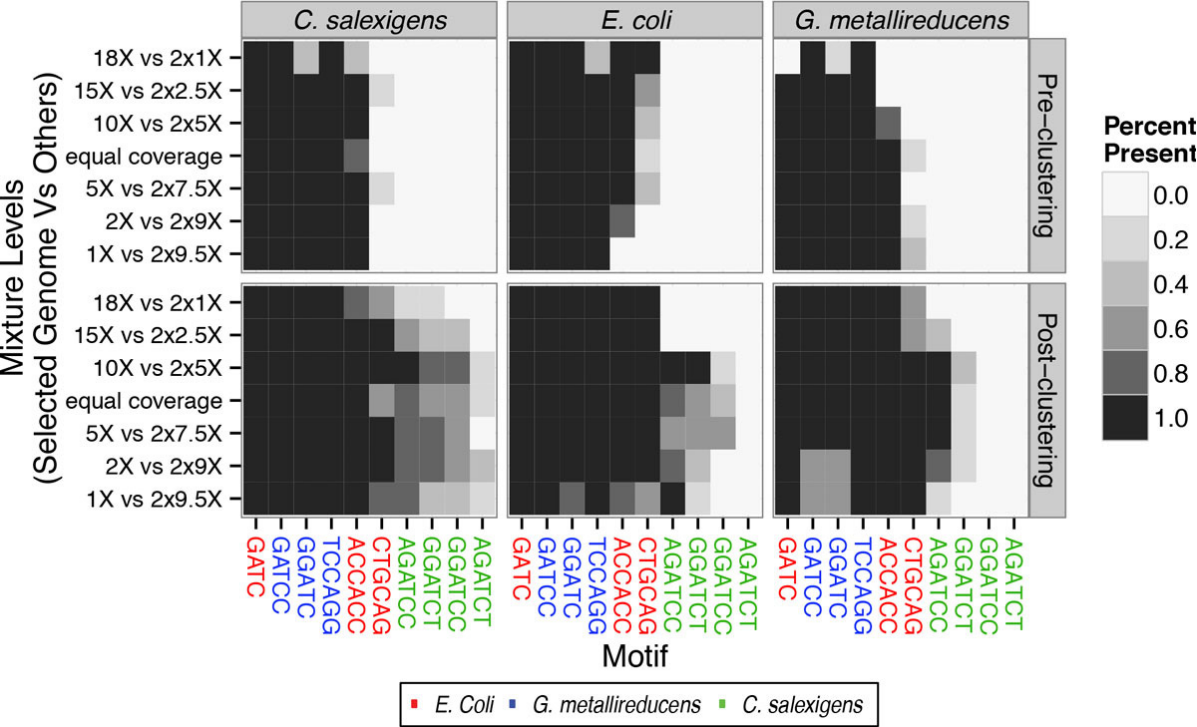
**True motifs observed from mixing genomes**. Each subplot corresponds to results when varying the corresponding genome (and keeping the remaining two genomes at equal coverage). The y-axis corresponds to different mixture levels, and the x-axis to each motif (with motifs colored by their genome of origin). Gray-scale gradient corresponds to detection rate over 5 simulations. Top plot is Pre-clustering motif detection, bottom Post-clustering motif detection.

Each simulated metagenomic dataset was then analyzed for significant motifs pre- and post-clustering. In the pre-clustering phase the same motif detection algorithm was run on the mixed samples as was run on the single genomes, except using the union of all reads and genomic sequences as input. Figure 4 (top) highlights the true motifs that are detectable at differing mixing levels, pre-clustering.

The pre-clustering results are surprisingly consistent across coverage levels. As expected, certain motifs ("ACCACC", "CTGCAG", and TCCAGG) are sometimes missed when their corresponding genome occur at low abundances. However, at all coverage levels *none *of the *C. salexigens *motifs are detectable. This is perhaps, not unexpected as all of its motifs are children of "GATC", "GGATC", and "GATCC". Given that they are all the same size (6) they most likely broaden the neighborhood distribution making it difficult to define them as outliers. This is problematic even when *C. salexigens *is at high-coverage because the shorter "GATC", "GATCC" and "GGATC" (in addition to being parents of the *C. salexigens *motifs) have more occurrences, thus allowing small differences in their distributions to have high LLRs. Additionally, as expected from the single genome simulations, the number of false positives was low (and often non-existent) across all simulations.

**Figure 5:**
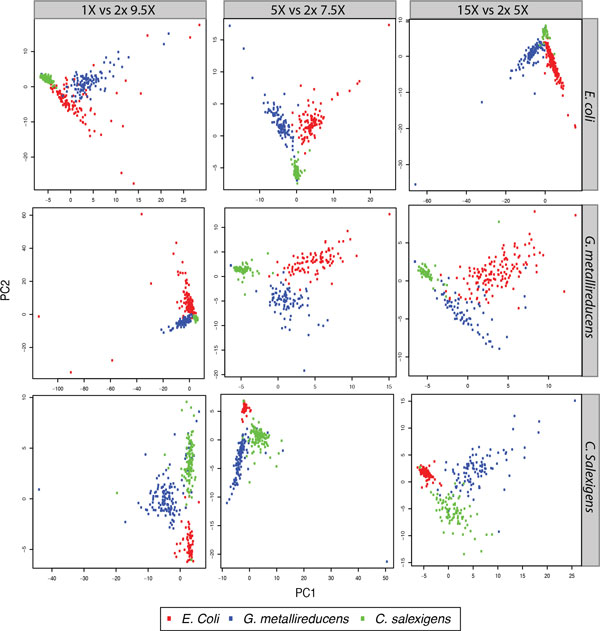
**PCA plot of contig vectors**. PCA plots for contig vectors representing the mean native vs. WGA IPD ratio for the set of significant motifs returned pre-clustering. Representative PCAs were chosen across multiple coverage profiles. Contigs are colored by their top genomic mapping hit via NUCmer [36].

These issues are partially resolved post-clustering, as shown in Figure 4 (bottom). In our simulation we computed motif vectors for all contigs greater than 10 kb. Figure 5 shows example PCA analysis (plotting for the first two components) at different coverage combinations of the contigs. Though contig separation is certainly cleaner at more even coverage levels, even at highly skewed coverages the contigs are largely spatially separated according to their constituent genomes. In most coverage senarios, three out of the four true *C. salexigens *motifs are able to be resolved, though "AGATCT" is consistently missed (Figure 4). Additionally, nearly all other motifs for the other two genomes are detectable in 100% of simulated datasets, across the full spectrum of coverages.

## Discussion

As single molecule sequencing becomes more common for epigenetic and assembly applications, approaches which take advantage of its unique features are increasingly necessary. Our approach builds on earlier studies to allow applications that were previously incompletely addressed: low coverage sequencing and metagenomics. On clonal genomes, we are able to detect most motifs at extremely low-coverage (0.1X), enabling the potential for identifying methylation motifs across a large number of genomes at extremely low-cost. In metagenomic simulations, we recover nearly all motifs at lower total coverage than is typically used for a single genome (20X) even when the genomes have highly disparate coverage profiles. Additionally, we show that these motifs provide an unbiased feature set for clustering contigs, potentially enabling further improvement to current metagenomic assembly and annotation approaches.

Some of the results shown are not immediately intuitive. For example, in Figure 4 post-clustering the metagenomics algorithm seemingly outperforms the single genome (Figure 3) scenario at the corresponding levels of coverage and contamination. The success of the two-step metagenomics pipeline is contigent on there being reasonable separation between contigs. This separation means that the clusters provide inputs that are relatively low in contamination when iterating the motif calling algorithm on each cluster, leading to the high recall rate shown post-clustering in Figure 4 relative to Figure 3. However, even in the ideal coverage scenario there is still some contamination in each cluster. This small degree of contamination potentially leads to missing the weaker "AGATCT" motif as it is outcompeted by false positive parental motifs. Iteratively running the clustering algorithm on new sets of motifs, until convergence, could potentially mitigate this issue.

Despite these benefits, the proposed method has limitations over other kinetic variation approaches. First, it relies on genome-wide motif signals, making it unable to directly assess site-level epigenetic markers. Unlike previous studies we cannot indicate the fraction of motifs modified or differential sites of modification between related samples [3,4]; at best we can only return a confidence value for a motif relative to other motifs in the sample. Moreover, this approach cannot take advantage of the richer models of kinetic variation that have been suggested, such as the CRF [15]. Context effects beyond the kmer of interest have been shown to greatly impact the variation in IPDs [16]. For example, we do not highly rank the "GCAAGG" in *C. jejuni *81-176 at 10X or less coverage (Supplemental Figure 1, additional file 1), and only moderately rank it at full coverage. This suggests that specific motif contexts still may require high depth even with this method. Notably, if we reduce the gamma distribution cutoff threshold to 5% (pre-Bonferonni correction) this motif (along with 4 other true positives) are identified (with the addition of 3 false positives). Ideally, the kmer-based approach could be used to identify candidate motifs to be rescored at each positional occurrence using site-specific tests. This would incur lower multiple hypothesis correction penalties than typical site-specific tests while simultaneously reducing false positives in both low-coverage and metagenomic settings, especially since we are currently forced to employ highly stringent significance cutoffs as empirical fittings of data to gamma and normal distributions are not necessarily well-calibrated.

Outside of the kinetic variation literature, there are far more complex algorithms that could be employed in the motif detection phase. There is a long history of motif detection algorithms employing a diverse set of approaches, including expectation maximization (MEME [24]), Gibbs sampling (Consensus [25]), suffix and mismatch trees (Weeder [26] and MITRA [27]), and graph-theoretic strategies (cWinnower [28]). These approaches typically look for enrichment of sequence motifs, as opposed to evaluating windows around ranked (modified) positions as one expects when presented with IPD data. Due to these constraints most studies have followed the iterative IPD sorting and enrichment procedure discussed in the introduction. However, the recent MotifMaker tool has directly integrated branch and bound search into the epigenetic motif finding problem [29], which should permit very sensitive searches, and if coupled with the suggested clustering strategy, could potentially be applied in a metagenomic setting. By attempting to integrate some of these previous approaches, one could potentially eliminate many of the false positive 'parental' signals we observe and better distinguish between neighboring signals. Additionally, these strategies appear necessary when confronting more complex motifs, as discussed below.

Motifs employing degenerate bases are not directly interrogated in the current implementation. In both *C. jejuni *strains we struggle to detect most motifs. This can be partially be explained by their degenerate sequence motifs, leading us to pick up parental motifs instead of the true children motifs. Explicitly, examining motifs with degenerate bases could improve sensitivity in low-coverage scenarios where few observations of each constituent motif exist. Including all degenerate kmers would substantially increase the number of tests; accounting for all 15 possible IUPAC symbols creates $k\cdot 15^{k}$ new motifs - which may become intractable for larger kmers. In the case of "RGATCY" shown in Table 1, the current implementation separately detects each of the four cases: "AGATCC", "GGATCT", "GGATCC", and "AGATCT". Interestingly, the "AGATCT" motif typically does not pass our significance threshold (or is preempted by the parent motif, "GATC"). This suggests either a weaker signal for this motif or that this variant of the consensus motif is not as frequently methylated. The latter explanation could explain the rate of detection in the previous study (76.5% as shown in Table 1). More statistical rigor could be applied to fitting the neighborhood distributions. The normal distribution may not adequately represent the tails of the neighbor LLR distributions and other (chi-squared or gamma) distributions should be considered. Additionally, a more accurate approach for rescoring parents would be to greedily remove IPDs present in higher-scoring children motifs and recalculate LLRs. The improved specificity would come at the cost of increased computation time.

Additionally, dyad motifs, such as "CACCN_6_CTC" and "GAGN_6_GTGG" in *C. salexigens*[30], are not easily detected via single kmer approaches. Creating degenerate motifs for all reasonable sized gaps is computationally expensive. In practice, we observed that these motifs sometimes occur as false positives within the current study. Therefore, a two pass approach which flags short kmers (from 3-6) passing a coarse significance threshold and then examines such motifs for potential dyad signals if they do not pass the monad significance threshold could dramatically reduce the number of dyad comparisons and may improve our power to detect real modifications.

Though the current approach relies on mapping to known references (or de novo assembled contigs), these are not strictly necessary to perform motif analysis on metagenomic datasets. With perfect reads, kmer distributions could be constructed from the raw reads. In practice, high error-rates not only entail a mapping step but we must also enforce a window of exact match to obtain reliable IPDs. One alternative approach is to build a database of long kmers, *D*, of length $k_{D}$ and align the reads to these kmers (or a debruijn graph constructed on *D*) instead of contigs. This tiered kmer strategy would eliminate context effects (assuming $k_{D}$ is long enough) while permitting more sensitive detection of low proportion genomes that are not accurately assembled and allowing for resolution of kmers at contig boundaries. Recently, various error-correction approaches have arisen for Pacbio sequencing [31] that suggest that short reads could be potentially eliminated from the process altogether. However, currently it is still not as cost-effective to perform the high-depth metagenomic sequencing necessary to detect, and accurately define kmers for low proportion genomes using solely SMRT sequencing. Thus, hybrid assembly approaches seem more pragmatic [32-34].

## Conclusion

Clear benefits exist for integrating this SMRT sequencing data and the proposed analysis approaches into existing metagenomics pipelines. Assembly from current metagenomics pipelines allows for a set of reference contigs to map raw reads and enumerate motifs. Annotation pipelines that provide phylogenetic information, such as MEGAN [35], could aid in the clustering of contigs for motif detection. Analogously, the long reads associated with SMRT sequencing along with the motif detection algorithm we present here, have the potential to substantially reduce contig fragmentation and improve clustering of contigs (especially in the case of novel genomes with poor annotation). Beyond extending current pipelines, our method provides a framework for using epigenetic profiles as an alternative metric for metagenomics sample comparison.

## Competing interests

A.B. has previously been employed at Pacific Biosciences (2009-2011).

## Authors' contributions

NB and SK assisted in design of the study, implemented part of the analysis pipeline, ran all simulations, and helped draft the manuscript. GF assisted in the design of the study and assisted in statistical analysis and interpretation of epigenetic signals. AB conceived of the study, participated in its design and coordination, implemented part of the analysis pipeline, and helped to draft the manuscript. All authors read and approved the final manuscript.

## Supplementary Material

Additional file 1 Supplementary materials Click here for file

## Declarations

Publication costs for this manuscript were funded in part by RECOMB-Seq and in part by the Icahn School of Medicine at Mount Sinai through seed funding to A.B.

This article has been published as part of BMC Bioinformatics Volume 15 Supplement 9, 2014: Proceedings of the Fourth Annual RECOMB Satellite Workshop on Massively Parallel Sequencing (RECOMB-Seq 2014). The full contents of the supplement are available online at http://www.biomedcentral.com/bmcbioinformatics/supplements/15/S9.
