# Supplementary material for: Detecting epigenetic motifs in low coverage and metagenomics settings
Source: BMC Bioinformatics. 2014 Sep 10;15(Suppl 9):S16. doi: 10.1186/1471-2105-15-S9-S16 (PMC4168715; doi:10.1186/1471-2105-15-S9-S16)
Supplement: Additional file 1 — Supplementary materials [file 1471-2105-15-S9-S16-S1.pdf]

Supplementary materials

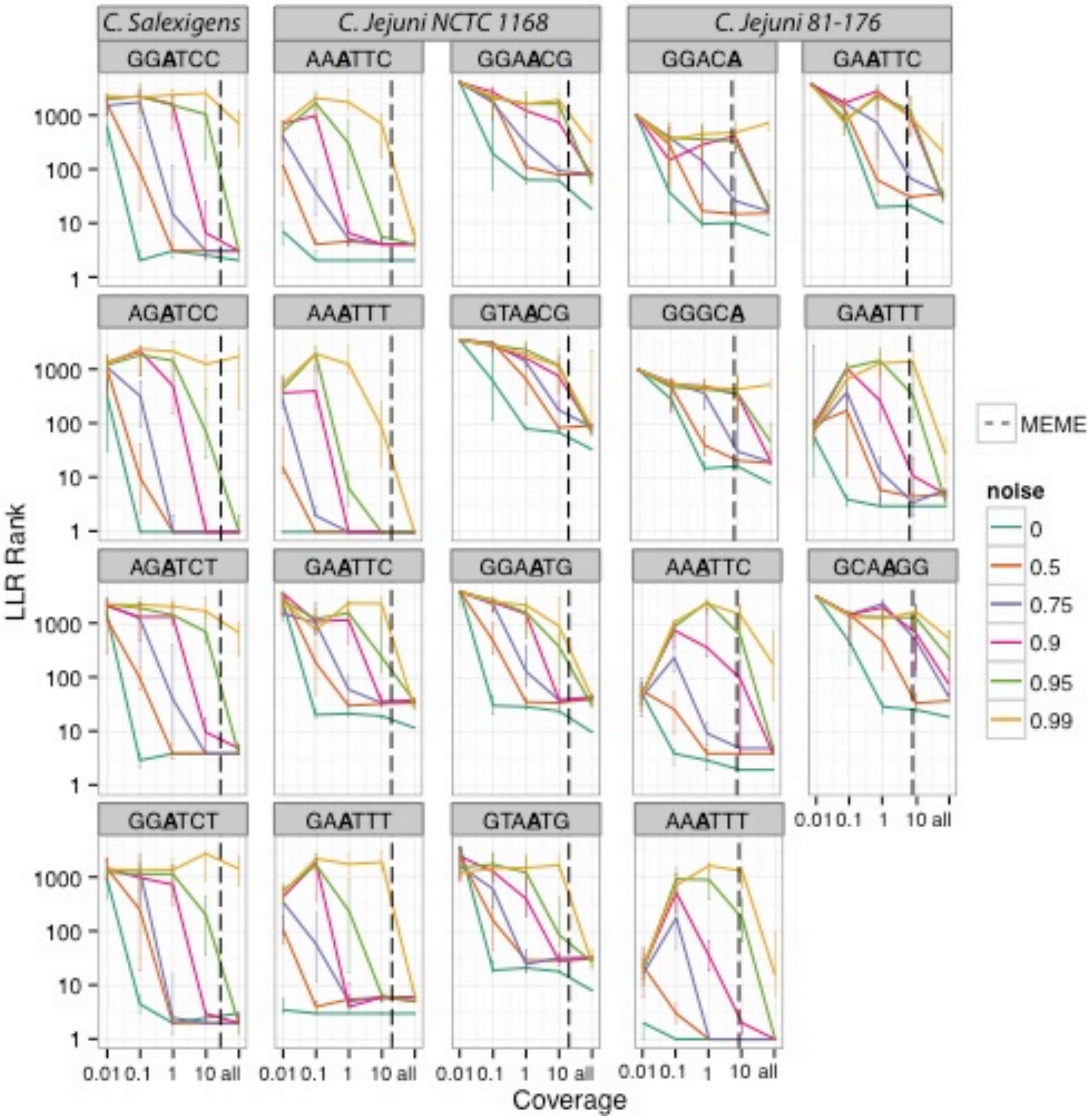

Supplemental Figure 1 - LLR ranking of true Kmers

Each kmer is ranked by its LLR score for multiple coverages and levels of "noise" (colors). The x-axis represents the coverage after downsampling (where "all" represents the entire dataset) and the y-axis the rank of the associated LLR value. Each point represents the median of 10 simulations, with upper and lower quartile bounds given by error-bars. Vertical black dashed lines correspond to the lowest coverage in which a majority of MEME runs (5 iterations) detect *any* true motif for the corresponding genome. The modified position per kmer is highlighted in each subplot title. All 5-mer true motifs and their children have been eliminated prior to running the 6-mer simulations.

Supplemental Table 1 – Run time and Memory Analysis

Input Dataset	Total Used IPDs (Millions)		LLR Kmer Table Generation		Significant Motif Assignment	
	WGA	Native	CPU time (hrs)	Memory (Mb)	CPU time (s)	Memory (Mb)
E. coli	221	345	2.89	37,641	41	115
C. salexigens	354	351	2.96	41,181	29	117
G. metallireducens	264	160	2.24	29,024	40	118
B. cereus	300	268	3.05	39,381	<1	28
C. jejuni NCTC	331	346	2.19	44,484	2	53
C. jejuni 81-176	339	201	1.87	30,764	14	113
Metagenomics Simulation (Avg.)	78-102	78-102	4.47	54,331	30	114

Memory and run time analysis for various stages of the pipeline. Alignment of sequencing data and conversion from PacBio cmp.h5 format are not included in analysis, since raw sequencing input files were unavailable from some studies.
